# Carglumic acid enhances rapid ammonia detoxification in classical organic acidurias with a favourable risk-benefit profile: a retrospective observational study

**DOI:** 10.1186/s13023-016-0406-2

**Published:** 2016-03-31

**Authors:** Vassili Valayannopoulos, Julien Baruteau, Maria Bueno Delgado, Aline Cano, Maria L. Couce, Mireia Del Toro, Maria Alice Donati, Angeles Garcia-Cazorla, David Gil-Ortega, Pedro Gomez-de Quero, Nathalie Guffon, Floris C. Hofstede, Sema Kalkan-Ucar, Mahmut Coker, Rosa Lama-More, Mercedes Martinez-Pardo Casanova, Agustin Molina, Samia Pichard, Francesco Papadia, Patricia Rosello, Celine Plisson, Jeannie Le Mouhaer, Anupam Chakrapani

**Affiliations:** Reference Centre for Inherited Metabolic Disorders (MaMEA) and IMAGINE Institiute, Necker-Enfants Malades Hospital, 149 Rue de Sevres, 75743 Paris, Cedex 15 France; Reference Center for Metabolic Diseases, Pediatric Neurology & Metabolic diseases, Robert Debre University Hospital, Paris, France; Unidad de Nutrición y Metabolismo, Hospital Infantil Sevilla, Sevilla, Spain; Centre de Référence des Maladies Héréditaires du Métabolisme, CHU Timone Enfants, Marseille, France; Complejo Hospitalario Universitario de Santiago de Compostela (CHUS), Santiago de Compostela, Spain; Servicio de Neurologíia Infantil, Hospital Vall d’Hebrón, Barcelona, Spain; Reference Center for Inherited Metabolic and Muscular Disease AOU, Meyer, Firenze, Italy; Hospital Sant Joan de Déu and CIBER-ER, Instituto de Salud Carlos III, Barcelona, Spain; Hospital Universitario Virgen de la Arrixaca, Murcia, Spain; Hospital Clínico Universitario de Salamanca, UCI Pediátricia, Salamanca, Spain; Reference Centre of Inherited Metabolic Disorders, Femme Mère Enfant Hospital, Lyon, France; Department of Metabolic Diseases, Wilhelmina Children’s Hospital, Utrecht, The Netherlands; Department of Pediatric Metabolism and Nutrition, Ege University Medical Faculty, Izmir, Turkey; Hospital Infantil Universitario La Paz, Madrid, Spain; Unidad Enfermedades Metabólicas Servicio de Pediatria, Hospital Universitario Ramón Y Cajal Ctra, Madrid, Spain; Unidad de Cuidados Intensivos Pediáticos, Hospital Clinic Universitario de Valencia, UCI Neonatal, Valencia, Spain; UOC of Metabolic and Genetic Diseases, Children’s Hospital Giovanni XXIII, Bari, Italy; Medical Affairs, Orphan Europe, Paris, France

**Keywords:** Carglumic acid, Hyperammonaemia, Organic acidurias (OAs), OA decompensation episodes

## Abstract

**Background:**

Isovaleric aciduria (IVA), propionic aciduria (PA) and methylmalonic aciduria (MMA) are inherited organic acidurias (OAs) in which impaired organic acid metabolism induces hyperammonaemia arising partly from secondary deficiency of N-acetylglutamate (NAG) synthase. Rapid reduction in plasma ammonia is required to prevent neurological complications. This retrospective, multicentre, open-label, uncontrolled, phase IIIb study evaluated the efficacy and safety of carglumic acid, a synthetic structural analogue of NAG, for treating hyperammonaemia during OA decompensation.

**Methods:**

Eligible patients had confirmed OA and hyperammonaemia (plasma NH_3_ > 60 μmol/L) in ≥1 decompensation episode treated with carglumic acid (dose discretionary, mean (SD) first dose 96.3 (73.8) mg/kg). The primary outcome was change in plasma ammonia from baseline to endpoint (last available ammonia measurement at ≤18 hours after the last carglumic acid administration, or on Day 15) for each episode. Secondary outcomes included clinical response and safety.

**Results:**

The efficacy population (received ≥1 dose of study drug and had post-baseline measurements) comprised 41 patients (MMA: 21, PA: 16, IVA: 4) with 48 decompensation episodes (MMA: 25, PA: 19, IVA: 4). Mean baseline plasma ammonia concentration was 468.3 (±365.3) μmol/L in neonates (29 episodes) and 171.3 (±75.7) μmol/L in non-neonates (19 episodes). At endpoint the mean plasma NH_3_ concentration was 60.7 (±36.5) μmol/L in neonates and 55.2 (±21.8) μmol/L in non-neonates. Median time to normalise ammonaemia was 38.4 hours in neonates vs 28.3 hours in non-neonates and was similar between OA subgroups (MMA: 37.5 hours, PA: 36.0 hours, IVA: 40.5 hours). Median time to ammonia normalisation was 1.5 and 1.6 days in patients receiving and not receiving concomitant scavenger therapy, respectively. Although patients receiving carglumic acid with scavengers had a greater reduction in plasma ammonia, the endpoint ammonia levels were similar with or without scavenger therapy. Clinical symptoms improved with therapy. Twenty-five of 57 patients in the safety population (67 episodes) experienced AEs, most of which were not drug-related. Overall, carglumic acid seems to have a good safety profile for treating hyperammonaemia during OA decompensation.

**Conclusion:**

Carglumic acid when used with or without ammonia scavengers, is an effective treatment for restoration of normal plasma ammonia concentrations in hyperammonaemic episodes in OA patients.

## Background

Organic acidurias (OAs) are a group of rare inherited diseases characterised by the excretion of non-amino organic acids in the urine. They result from the impaired metabolism of organic acids, leading to abnormal and toxic levels of organic acids in the blood, urine and tissues, causing serious health problems [1, 2]. Three classical OAs have been identified, namely isovaleric aciduria (IVA), propionic aciduria (PA) and methylmalonic aciduria (MMA). IVA is caused by a deficiency of isovaleryl-CoA dehydrogenase, resulting in defective breakdown of leucine. PA occurs due to a deficiency of propionyl-CoA carboxylase and MMA is caused by a defect in the conversion of methylmalonyl-CoA to succinyl-CoA, both of which affect the metabolism of amino acids isoleucine, valine, methionine and threonine [1].

**Fig. 1:**
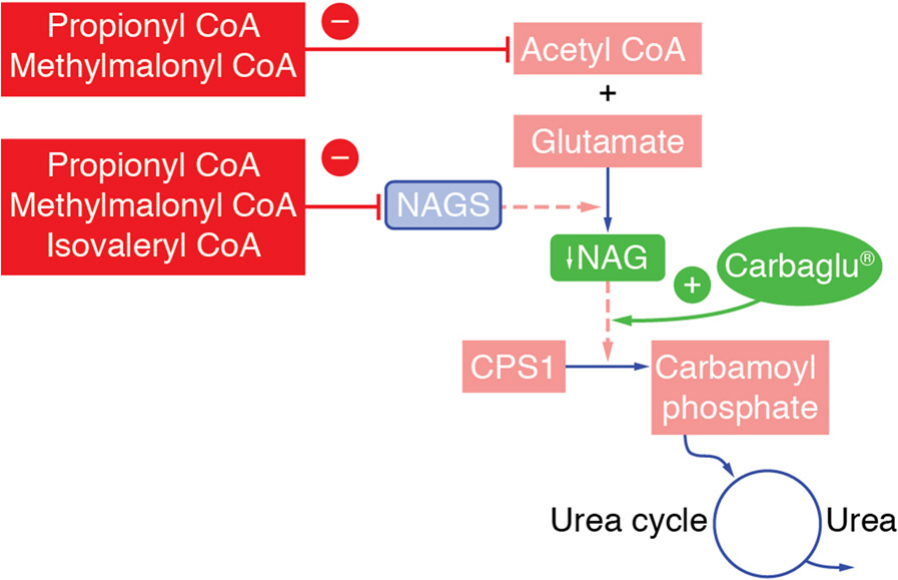
Pathophysiology of hyperammonaemia in organic acidurias and action of carglumic acid. CoA, coenzyme A; CPS-I, carbamoyl-phosphate synthase I; NAG, N-acetylglutamate; NAGS, N-acetylglutamate synthase; -, inhibition; +, activation

OAs usually develop in the neonatal period, characterised by toxic encephalopathy within the first few days of life, with symptoms including vomiting, poor feeding, seizures and abnormal tone, and lethargy progressing to coma [3, 4]. This metabolic decompensation is a medical emergency that can be fatal or result in brain damage if patients do not receive immediate medical interventions [2]. Mechanisms for acute encephalopathy include direct neurotoxicity of metabolites and hyperammonaemia [1, 5]. Normally the level of ammonia is controlled by conversion to urea in the liver and subsequent excretion in urine. In the classical OAs, propionyl CoA, methylmalonyl CoA and isovaleryl CoA accumulate and inhibit N-acetylglutamate (NAG) synthase (NAGS). NAGS catalyses the formation of NAG, an activator required by carbamoyl-phosphate synthetase I (CPS-I). CPS-I is a key enzyme of ureagenesis that catalyses the first and rate-limiting step of the urea cycle [6–9]. Propionyl CoA and methylmalonyl CoA also inhibit the pathway by depleting hepatic acetyl CoA, which is required for NAG synthesis. Figure 1 provides an overview of the mechanism of hyperammonaemia in OAs.

Reducing ammonia levels as quickly as possible is one of the medical priorities in acute decompensation episodes of OAs as a longer duration and higher levels of hyperammonaemia are associated with worse neurological outcomes [10]. First-line management includes the promotion of anabolism through the administration of high calorie energy sources, such as intravenous glucose and a protein-restricted diet [7]. Ammonia scavengers such as sodium benzoate and sodium phenylbutyrate (excreted as hippurate and phenylacetylglutamine after conjugation with glycine or glutamine, respectively [11, 12]) have a higher renal clearance than ammonia itself, and are therefore used for accelerating ammonia elimination in the urine [7]. Haemodialysis or peritoneal dialysis may be started if these interventions do not generate significant reductions in blood ammonia levels within a few hours.

Carglumic acid is a synthetic structural analogue of NAG, and as such is a specific intervention for hyperammonaemia in these patients. This was approved as Carbaglu® by the European Medicines Agency (EMA) for the treatment of hyperammonaemia due to primary NAGS deficiency, and hyperammonaemia due to IVA, MMA and PA [13]. Carglumic acid accelerates ammonia detoxification by mimicking the effects of NAG on CPS-I, thereby driving the urea cycle forward independent of other mechanisms for detoxifying the organic acids (Fig. 1). In the first published clinical study, Ah Mew et al. showed that single dose/short-term treatment with carglumic acid was associated with reduced ammonia and glutamine levels and an increase in ureagenesis [8].

Carglumic acid has been used in humans since 1981 in the acute and long-term treatment of NAGS deficiency. Although a number of publications describe the response to, and safety of carglumic acid in the treatment of hyperammonaemia due to OA decompensation episodes or NAGS primary deficiency [7, 8, 14, 15], carglumic acid’s orphan drug status highlights the need for further research into its effectiveness in treating OA-induced hyperammonaemia in various age groups and in patients with different aetiologies of hyperammonaemia.

This retrospective, multicentre, open-label, uncontrolled, phase IIIb study was designed to evaluate the efficacy and safety of carglumic acid in OA patients with hyperammonaemia during metabolic decompensation episodes. The primary objective of this study was to study the change in plasma ammonia levels as the main biological response to carglumic acid treatment during episodes of hyperammonaemia in OA. Secondary objectives include describing the demographic profile of the patient population, evaluating the clinical and biological responses to treatment and assessing the safety of carglumic acid.

## Ethics, consent and permissions

This study was conducted in accordance with Good Clinical Practice, the Principles of the Declaration of Helsinki and the requirements of local ethics committees (Spain: Comité Etico de Investigacion Clinica de Aragon, Avda. San Juan Bosco, Zaragoza; Comité Etico de Investigacion Clinica Hospital Clinico Universitarion de Valencia, Avda. Vicente Blasco Ibanez, Valencia. France: Comité de Protection des Personnes Ile De France II, Centre Universitaire des Saints-Pères, rue des Saints-Pères, Paris. Italy : Comitato Etico dell Azienda, Ospedaliera Policlinico Consorziale di Bari; Comitato Etico Locale per la sperimentazione dei farmaci, dell’azienda ospedaliero-Universitaria Anna Meyer de Firenze. UK: Leicestershire, Northamptonshire and Ruland Ethics Committee 2, Nottingham. Germany: Ethik-kommission des Fachbereichs Medizin der Johann Wolfgang Goethe-Universitat, Frankfurt. Netherlands: Stichting CGR Hanzeweg, Goud.)

Informed consent was obtained for each patient prior to commencement of the planned data collection, review and analysis. Cases where circumstances made it necessary to waive informed consent (eg if the patient was dead or lost to follow-up) were dealt with on a case by case basis and in accordance with local regulations.

## Methods

Data were collected from 21 centres in Italy, France, Germany, the Netherlands, Spain, Turkey and the United Kingdom from patients treated between January 1995 and October 2009.

### Patient eligibility

Eligible patients had a confirmed diagnosis of OA with hyperammonaemia (defined as plasma NH_3_ concentration above 60 μmol/L before carglumic acid treatment) during at least one full OA decompensation episode that was treated with carglumic acid. Patients with severe hepatic insufficiency at the time of carglumic acid treatment, inherited hepatic malformation, or intercurrent disease (other than OA) that might have generated hyperammonaemia were excluded from the study.

### Study treatment

As a retrospective, observational study, dosing regimens were not predefined. The recommended initial dose of carglumic acid in Europe is 100–250 mg/kg/day, administered orally twice or thrice daily. Carglumic acid doses in this study were at the discretion of the treating physician, as was the duration of treatment. However, the evaluation window was defined as up to a maximum of 15 days from the first administration of carglumic acid. Some patients were treated with ammonia scavenger medications, including sodium benzoate and sodium phenylbutyrate, prior to and/or concomitantly with carglumic acid.

### Study evaluations and endpoints

All available retrospective efficacy and safety data were collected for each OA decompensation with hyperammonaemia episode. A single episode of hyperammonaemia was defined as an acute episode lasting up to 15 days (defined based on expert opinion and previous experience and assessed by the investigators based on both ammonia levels and clinical symptoms). If treatment with carglumic acid was discontinued before Day 15 and there was a >20 % increase of plasma ammonia compared with the last measurement that occurred beyond 24 hours after discontinuation, this was considered a new episode. If carglumic acid treatment was continued after Day 15, a >20 % increase of plasma ammonia compared with the last measurement was recorded as a new episode. Efficacy measurements were assessed at baseline, defined as the evaluation just prior to initiation of carglumic acid treatment for each episode, and at the study endpoint, defined as end of treatment (no more than 18 hours after the last intake of carglumic acid) or the last available value under treatment up to a maximum of 15 days.

The primary evaluation was plasma NH_3_ concentration at the study endpoint. Normalisation of plasma NH_3_ concentration was defined as plasma NH_3_ concentration ≤60 μmol/L. Secondary evaluations at study endpoint included other biological markers (plasma amino acids, plasma and urinary organic acids and blood biochemistry), clinical markers of neurological, psychiatric, psychomotor, respiratory and hepatic status, and safety assessments. Time to reach plasma NH_3_ ≤ 60 μmol/L was also assessed.

### Statistical analysis

Analysis was carried out on episodes of hyperammonaemia in the efficacy population, as a single patient could have more than one episode. The efficacy population included all enrolled patients who received at least one dose of carglumic acid and had a baseline and endpoint plasma NH_3_ measurement for the same episode. Patients or episodes were excluded from the efficacy analyses if there was one or more major protocol deviation such as missing NH_3_ data at baseline or endpoint, unconfirmed diagnosis or deviations relating to the inclusion/exclusion criteria.

Descriptive analyses were performed, with continuous variables summarised by descriptive statistics (number of patients [n], mean, standard deviation, minimum, median and maximum) and categorical data presented by absolute and relative frequencies (n and %). Unless otherwise indicated, summary statistics were reported for observed data only.

The primary evaluation was analysed using a repeated measures analysis of change from baseline in plasma NH_3_ concentration at study endpoint for all episodes. The least squares mean change and 95 % confidence intervals (CI) are reported. Missing data were not imputed. Secondary evaluations were analysed in a similar manner. Subgroup analyses of the primary and secondary evaluations were performed, including OA type, neonate (≤28 days of age at start date of carglumic acid treatment) vs non-neonate and use of ammonia scavenger medications and/or haemodialysis/haemofiltration.

The safety population was defined as all patients exposed to at least one dose of study drug; safety analyses comprised incidence of treatment-emergent adverse events (TEAEs), clinical laboratory parameters and concomitant medications. Drug-related AEs were defined as those considered by the investigator as being either related to use of the study drug or of unknown causality.

## Results

### Patient disposition

**Fig. 2:**
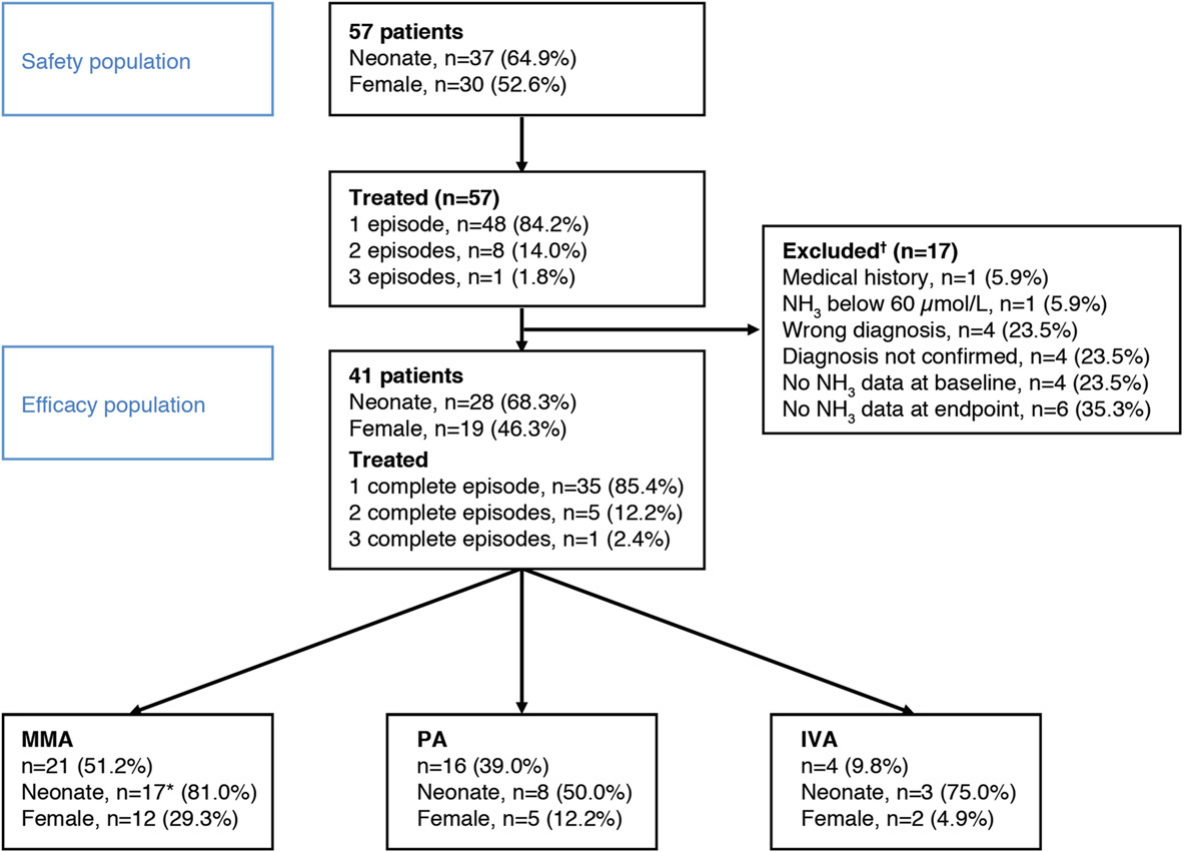
Participant flow. *Includes one patient treated twice as a neonate. ^†^Some cases excluded for multiple reasons. IVA, isovaleric aciduria; MMA, methylmalonic aciduria; NH_3_, ammonia; PA, propionic aciduria

Fifty-seven patients, of whom 64.9 % were neonates, were included in the study and constituted the safety population; most of these were treated for a single episode of hyperammonaemia (48 patients; 84.2 %; Fig. 2). Seventeen patients had major protocol deviations, resulting in exclusion from the efficacy analyses; however one patient had a protocol deviation for one of the two recorded episodes, and thus remained in the efficacy population. Of the 41 patients in the efficacy population, 35 had a single recorded episode of hyperammonaemia treated with carglumic acid (Fig. 2). The remaining episodes within the efficacy population were experienced by five patients who had two episodes and one patient who had three episodes.

Additional management with a low protein diet was recorded in 73 % of the 26 episodes with available data at baseline and 59 % of the 39 episodes with available data at study endpoint. Furthermore, treatment with at least one of L-carnitine, L-glycine or L-arginine, before and/or during treatment with carglumic acid, was administered in 38 and 58 episodes within the efficacy and safety populations, respectively.

### Episode characteristics in the efficacy population

Of the 48 hyperammonaemia episodes included in the efficacy analysis, 29 occurred in neonates and 19 in non-neonates. MMA and PA were the most common OA diagnoses (Fig. 2). The mean duration of episodes ranged from 5.0 to 9.8 days for the different OAs and was shorter in neonates compared with non-neonates (Table 1). The median age at presentation with a study episode was 0.2 and 12.7 months for neonates and non-neonates, respectively.

**Table 1:** Episode characteristics by patient diagnosis in the efficacy population

	Isovaleric aciduria	Methylmalonic aciduria	Propionic aciduria	Neonates	Non-neonates	Total
N, patients	4	21	16	28	13	41
Gender						
Male	2	9	11	14	8	22
Female	2	12	5	14	5	19
N, episodes	4	25	19	29^a^	19	48
Median age at episode, days	7 (neonate)	5 (neonate)	6.5 (neonate)	6	387	6.0 (neonate)
						387.0 (non-neonate)
Median age at episode, months	44.84 (non-neonate)	2.53 (non-neonate)	12.73 (non-neonate)	0.20	12.73	0.2 (neonate)
						12.73 (non-neonate)
Duration of episode,						
Mean, days (SD)	5.0 (1.8)	7.8 (6.0)	9.8 (10.5)	6.6 (4.8)	11.0 (10.8)	8.4 (7.9)
Median, days	5.0	7.0	6.0	6.0	7.0	6.0
Range, days	3–7	2–26	2–43	2–20	3–43	2–43
Concomitant treatment^b^ (%)	0 (0.0)	12 (48.0)	9 (47.4)	18 (62.1)	3 (15.8)	21 (43.8)
Scavengers	0	9	6	13	2	15
Haemodialysis/haemofiltration	0	3	3	5	1	6
Baseline NH_3_, μmol/L						
Mean (SD)	666.8 (692.2)	296.9 (206.2)	355.0 (326.8)	468.3 (365.3)	171.3 (75.7)	350.7 (321.3)
Median	435.0	247.8	213.0	328.0	164.0	215.0
Range	164.0–1633.0	76.1–868.0	76.0–1200.0	96.0–1633.0	76.0–385.0	76.0–1633.0

^a^One patient was treated for two episodes of hyperammonaemia within 28 days of birth

^b^Concomitant treatment includes ammonia scavengers and haemodialysis/haemofiltration

SD, standard deviation

Twenty-one of the 48 hyperammonaemia episodes (44 %) in the efficacy population were treated with additional ammonia lowering therapy, such as ammonia scavengers and haemofiltration, before or during carglumic acid treatment. Similar patterns of use were seen in the MMA and PA efficacy populations, although none of the IVA episodes were treated with additional ammonia lowering therapy. Use of additional ammonia lowering therapy was higher in neonates compared to non-neonates (Table 1).

Carglumic acid treatment was discontinued in the majority of cases due to the patient reaching the end of an episode (86.7 %). Discontinuation due to death and other reasons such as a dramatic decrease of ammonia plasma concentration, adequate effect with other drugs and normalisation of ammonaemia were seen in 4.4 % and 8.9 % of episodes, respectively. No differences in the reasons for discontinuation of carglumic acid were noted between neonates and non-neonates.

### Ammonia profile in response to carglumic acid treatment

**Fig. 3:**
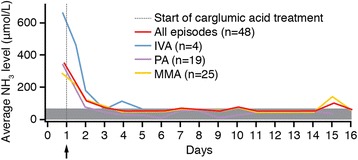
Ammonia profile in response to carglumic acid treatment. Endpoint NH_3_ in response to treatment with Carbaglu® for each of the diagnostic groups was 52.5 μmol/L (IVA), 67.7 μmol/L (MMA) and 47.8 μmol/L (PA). The time to achieve endpoint was equivalent for the three groups. IVA, isovaleric aciduria; MMA, methylmalonic aciduria; PA, propionic aciduria. Analysis by episode for the total efficacy population and for diagnosis subpopulations

Following initiation of carglumic acid treatment, the high average baseline levels of plasma NH_3_ (Table 1) decreased rapidly to levels close to the normalisation level of 60 μmol/L by Day 3 of treatment in all three OA subgroups (Fig. 3). Generally plasma NH_3_ levels were maintained over the two week follow-up period. Similar mean endpoint plasma NH_3_ concentrations were seen when summarised by diagnostic subgroup, ie MMA, 67.7 ± 36.4 μmol/L; IVA, 52.5 ± 28.4 μmol/L; PA, 47.8 ± 20.4 μmol/L.

**Fig. 4:**
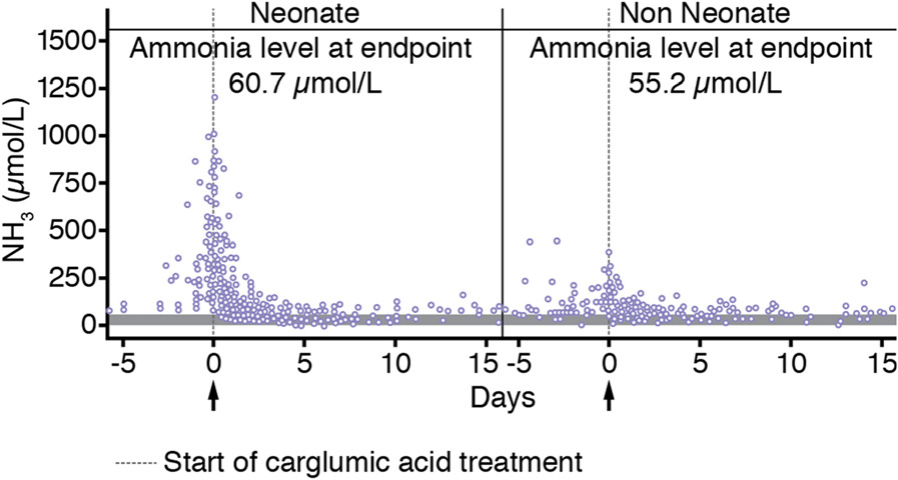
Ammonia levels in response to carglumic acid therapy by age category.* Ammonia levels in the neonate group were higher than those in the non-neonate group at baseline; however, both groups achieved a similar endpoint ammonia level within the same timeframe (18 hours). *Analysis by episode in the efficacy population; shaded grey area is target plasma ammonia concentration

The endpoint mean plasma NH_3_ concentration was 60.7 ± 36.5 μmol/L for neonates and 55.2 ± 21.8 μmol/L for non-neonates (Fig. 4). The primary evaluation of mean change in plasma NH_3_ concentration from baseline to endpoint for the total efficacy population revealed a mean (95 % CI) reduction of -292.2 (-385.4 to -199.0) μmol/L. The mean changes in plasma NH_3_ concentration from baseline to endpoint for the different subgroups are given in Table 2, while the mean changes in plasma NH_3_ from baseline to achievement of a normalised ammonia level (≤60 μmol/L) are provided in Table 3.

**Table 2:** Change in plasma ammonia concentration from baseline to study endpoint in efficacy population

	Episodes *n*	Mean change in NH_3_ at endpoint (μmol/L)	Mean time to the endpoint, days
Total	48	-292.2	4.6
Isovaleric aciduria	4	-614.3	2.8
Methylmalonic aciduria	25	-229.2	5.2
Propionic aciduria	19	-307.3	4.2
Neonates	29	-407.6	4.0
Non-neonates	19	-116.1	5.6
With concomitant ammonia scavengers	15	-366.0	3.9
Without concomitant ammonia scavengers/haemofiltration	27	-200.2	5.0
With haemofiltration (with or without ammonia scavengers)	6	-521.8	4.8

NH_3_, ammonia

**Table 3:** Change in plasma ammonia concentration from baseline to achievement of normalised plasma ammonia^ab^

	Episodes n	Mean change at NH_3_ ≤ 60 μmol/L	Mean time to achieve NH_3_ ≤ 60 μmol/L, days
Total	42	-272.8	2.4
Isovaleric aciduria	3	-299.0	2.3
Methylmalonic aciduria	20	-233.1	1.9
Propionic aciduria	19	-310.5	3.0
Neonates	24	-392.6	2.0
Non-neonates	18	-113.2	3.1
With concomitant ammonia scavengers	14	-344.7	2.3
Without concomitant ammonia scavengers	22	-157.2	2.8
With haemofiltration (with or without ammonia scavengers)	6	-529.3	1.5

^a^Normalised plasma ammonia defined as NH_3_ ≤ 60 μmol/L

^b^Efficacy population

NH_3_, ammonia

The median time to achieve normalisation of plasma NH_3_ concentration after initiation of carglumic acid treatment for the episodes treated with ammonia lowering therapy was 36.5 hours and without 37.4 hours. The median time to achieve NH_3_ ≤ 60 μmol/L among the three analysed OA subgroups was 40.5 hours for IVA; 37.5 hours for MMA; 36 hours for PA. The median time to achieve normalisation of plasma NH_3_ concentration was 38.4 hours in the neonate group and 28.3 hours in the non-neonate group. Concomitant use of haemodialysis/haemofiltration was associated with a higher rate of change in plasma NH_3_ concentration from baseline to study endpoint. The median baseline plasma NH_3_ level was 506.5 μmol/L in patients who received haemodialysis/haemofiltration compared to 206 μmol/L in patients who did not receive these treatments; at endpoint the median plasma NH_3_ levels were reduced to 31 μmol/L and 55.5 μmol/L, respectively. However the number of episodes treated with haemodialysis/haemofiltration was low (*n* = 6). It was noted that the episodes concomitantly treated with scavengers were the more severe cases associated with higher levels of plasma NH_3_ at baseline.

### Clinical symptoms

At baseline all 48 episodes were associated with clinical symptoms, including poor sucking, vomiting, muscle hypotonia, hyperventilation, lethargy, coma and recurrent ketoacidosis. At the end of the episodes with available baseline and endpoint data, there were no clinical symptoms in 8 of 30 (26.7 %) episodes. At baseline only 12 % of episodes (3/25 episodes with available baseline and study endpoint data) occurred in patients with normal neurological status compared with 52 % (13/25) of episodes at study endpoint. The most commonly observed neurological symptom was somnolence, the prevalence of which decreased from 68 % at baseline to 28 % at study endpoint.

A lower incidence of clinical symptoms was observed at study endpoint compared with baseline where data were available at both time points. Normal psychiatric status was recorded in 87.5 % (21/24) of episodes at study endpoint compared with 70.8 % (17/24) at baseline. Similarly, normal hepatic status and normal respiratory status were seen in 84.4 % (27/32) and 83.9 % (26/31) of episodes, respectively at study endpoint compared with 75.0 % (24/32) and 61.3 % (19/31) episodes, respectively at baseline. The incidence of normal psychomotor status was 64 % both at baseline and at study endpoint [16/25]).

### Safety evaluations

Overall, in this retrospective observational study in patients with OA, 25 of 57 patients in the safety population experienced at least one AE (Table 4). A total of 74 AEs were reported, including 23 severe AEs and 22 serious AEs (SAEs). The reported AEs were mainly from four system organ classes: general disorders and administration sites conditions (15.8 %), blood and lymphatic system disorders (14.0 %), infections and infestations (12,3 %), and metabolism and nutrition disorders (12.3 %). There were 11 SAEs in seven patients that resulted in death. These patients died from usual complications observed during episodes of decompensation in OA, such as cardiogenic shock, multi-organ failure, metabolic disorders and respiratory arrest.

**Table 4:** Summary of treatment-emergent adverse events in the safety population

	Events (%)	Patients (%)*n* = 57
Any AEs	74	25 (43.9)
All severe AEs	23 (31.1)	13 (22.8)
All serious AEs	22 (29.7)	13 (22.8)
All drug-related AEs^a^	24 (32.4)	9 (15.8)
All serious drug-related AEs^b^	6 (8.1)	5 (8.8)
All AEs leading to death	11 (14.9)	7 (12.3)

^a^AEs with an investigator’s causality assessment of either ‘related’ or ‘unknown’. Of these, none were assessed as ‘related’

^b^All serious drug-related AEs with an investigator assessment of ‘related’ or ‘unknown’. Of these, only one was assessed by the investigator as ‘related’ and this was neurological damage that resulted in death

AEs, adverse events

All these deaths were thought by the investigators to be unrelated to carglumic acid, except for in one patient who had neurological symptoms prior to study drug administration (poor sucking, muscle hypotonia, lethargy and somnolence). He died due to neurological damages and respiratory arrest six days after discontinuation of carglumic acid (given for nine days), and therefore the causality of neurological damage probably leading to death seems unlikely to be related to carglumic acid treatment.

### Treatment initiation, dosage and treatment duration

The mean time between the start of an episode and initiation of carglumic acid was 3 days for the total efficacy population (Table 5). For the individual diagnoses, the mean time to initiation of treatment was 0.8 (IVA), 2.5 (MMA) and 4.2 (PA) days. In neonates, the mean time from the start of an episode to the beginning of treatment was 1.5 days, while in non-neonates it was 5.3 days. For the efficacy population, the mean (SD) length of carglumic acid treatment was 5.5 (4.6) days. Mean (SD) length of treatment was 4.9 (3.5) days for neonates and 6.5 (5.9) days for non-neonates.

**Table 5:** Treatment initiation, dosage and treatment duration in the efficacy population

	Isovaleric aciduria (*n* = 4)	Methylmalonic aciduria (*n* = 25)	Propionic aciduria (*n* = 19)	Neonates(*n* = 29)	Non-neonates(*n* = 19)	Total(*n* = 48)
Time to treatment initiation						
Mean, days (SD)	0.8 (1.0)	2.5 (5.5)	4.2 (9.5)	1.5 (2.8)	5.3 (10.6)	3.0 (7.2)
Median (range)	0.5 (0–2)	0.0 (0–24)	2.0 (0–41)	1.0 (0–14)	1.0 (0–41)	1.0 (0–41)
First dose						
Mean, mg/kg (SD)	99.0 (63.3)	78.2 (64.9)	119.5 (83.2)	106.7 (75.3)	80.4 (70.5)	96.3 (73.8)
Median, mg/kg (range)	81.6 (50.0–182.7)	51.0 (13.7–236.2)	106.0 (13.3–303.0)	100.0 (13.7–303.0)	50.0 (13.3–256.4)	75.5 (13.3–303.0)
Initial 24-h dose						
Mean, mg/kg (SD)	216.0 (217.9)	160.6 (108.3)	209.3 (186.9)	214.1 (179.1)	139.2 (81.7)	184.5 (151.8)
Median, mg/kg (range)	131.6 (62.5–538.5)	129.0 (41.1–470.8)	190.2 (53.1–909.1)	170.2 (41.1–909.1)	100 (45.3–333.3)	156.4 (41.0–909.1)
Treatment duration						
Mean, days (SD)	3.5 (1.7)	6.1 (4.6)	5.2 (5.0)	4.9 (3.5)	6.5 (5.9)	5.5 (4.6)
Median, days (range)	3.5 (2–5)	5.0 (1–15)	3.0 (1–15)	4.0 (1–15)	3.0 (1–15)	4.0 (1–15)

*SD*, standard deviation

The mean first dose of carglumic acid for episodes in the efficacy population was 96.3 mg/kg. In neonates, the mean first dose of carglumic acid was 106.7 mg/kg, while in non-neonates, the mean first dose was 80.4 mg/kg. The first dose of carglumic acid in patients concomitantly treated with ammonia scavengers was 125.4 mg/kg (mean) and 106.4 mg/kg (median). Patients not treated with ammonia scavengers received a mean dose of 73.6 mg/kg (median: 50.0 mg/kg). However, a wide range of first dose sizes was administered in all groups (Table 5). The most common initial 24-hour dose category was 100–250 mg/kg, with the mean 24-hour carglumic acid across all patients being 184.5 mg/kg (Table 5). The initial daily dose of carglumic acid administered to the total efficacy population was consistent with the EMA label (100–250 mg/kg). Dose reductions during the course of treatment resulted in a shift from a predominance of doses in the 100–250 mg/kg range to a relatively even distribution of episodes treated with these doses and lower doses of 50–100 mg/kg and <50 mg/kg.

## Discussion

This study demonstrated that carglumic acid, with or without additional therapies such as ammonia scavengers, induced a rapid and effective reduction of plasma ammonia during decompensation episodes in OAs. It represents the largest body of evidence to date on the efficacy and safety of carglumic acid in the management of hyperammonaemia in patients with OAs. Mean reductions to similar ammonia levels at study endpoint were seen in patients with IVA, MMA and PA. Furthermore, carglumic acid treatment of hyperammonaemia episodes reduced plasma NH_3_ concentrations to similar levels in neonates and non-neonates, despite neonatal episodes having higher ammonia concentrations at treatment initiation. These data support the use of carglumic acid in the treatment of hyperammonaemia in neonates and non-neonates with any of the three classical OA types. Reductions in plasma NH_3_ concentration following carglumic acid treatment were accompanied by improvements in clinical symptoms and neurological status.

Mean plasma NH_3_ concentration at endpoint was similar in patients treated with scavengers compared to those not treated with scavenger medication (55.6 vs 60.8 μmol/L). However, mean baseline NH_3_ concentration was higher in the group of patients who received scavengers (466.1 vs 261.0 μmol/L). When the higher baseline NH_3_ level is considered, there was a higher rate of change in plasma NH_3_ levels in patients treated with scavengers (-410.5 vs -200.2 μmol/L). Reduction in plasma NH_3_ concentration was more rapid in episodes of hyperammonaemia treated with carglumic acid and scavengers compared with those not treated with scavengers. These analyses were repeated excluding one outlier, who experienced a very rapid and large decrease in ammonia levels, and yielded similar results. However, the hyperammonaemia episodes treated with scavengers were more severe at presentation and scavenger use was more frequent in neonates, thus the observed differences between these groups are unsurprising. As expected, the use of haemodialysis prior to or concomitantly with carglumic acid was also associated with a more rapid response. Although use of scavengers may be beneficial by increasing reduction of plasma NH_3_ levels, there are a number of potential disadvantages to be considered. Sodium phenylacetate may induce glutamine depletion as it conjugates with glutamine to yield phenylacetylglutamine, which is subsequently excreted in the urine [16]. Additionally, sodium phenylacetate and sodium benzoate may potentiate ammonia toxicity by blocking the urea cycle through sequestration of CoA [17, 18] and caution when used in organic acidemias was suggested by recent guidelines [19]. Moreover, treatment with these scavenging drugs at high doses may increase serum sodium and decrease serum potassium levels [18]. Consequently, measuring plasma levels of sodium benzoate is recommended in the neonatal period, particularly in jaundiced infants; however, this analysis is not available in most centres [18].

The response to carglumic acid with or without ammonia scavengers in neonates was faster than in non-neonates; however, it is difficult to separate the effects of scavenger treatment from the effects due to age. The rapidity of response in non-neonates was similar with or without scavengers; however, more data are needed as only two non-neonates were treated with scavengers in this study. The time to response following carglumic acid administration was similar in patients with MMA and PA. The small number of patients with IVA and the high plasma NH_3_ concentration at treatment initiation in these patients make it difficult to draw conclusions on the time to response compared with the other OAs.

The relatively small number of IVA cases in this study (*n* = 4) is consistent with the frequency observed in clinical practice. Clinical experience suggests that patients with IVA have less severe hyperammonaemia [5, 20] and are easier to manage compared to patients with other OAs.The higher baseline ammonaemia in the IVA episodes in this study suggests that only the most severe cases were treated with carglumic acid. In addition, this small sample population had an artificially high mean and median plasma NH_3_ concentration due to an outlier (1633 μmol/L). The larger number of neonates (*n* = 28) compared with non-neonates (*n* = 13) included in the study reflects the more severe hyperammonaemia seen in neonates at presentation [3]. Unsurprisingly, higher doses of carglumic acid were administered to neonates, as in general the more severe hyperammonaemia episodes were treated with high doses. Similarly episodes in neonates vs non-neonates were more frequently concomitantly treated with scavengers, as they were more severe cases with higher baseline plasma NH_3_ levels.

Dietary management is often used as first-line treatment in the management of decompensation episodes [19, 21]; however, the impact is limited as an extremely restricted diet cannot be maintained for more than 48 hours [19]. Carglumic acid treatment might allow a faster reintroduction of protein into the diet and thus limit muscular catabolism contributing to hyperammonaemia as seen in the context of NAGS deficiency [22].

The clinical presentation of patients with metabolic decompensation at baseline was consistent with other reports in the literature [19], with marked neurological and gastrointestinal symptoms as well as anaemia, ketosis and metabolic acidosis. Eight patients were comatose prior to treatment with carglumic acid. A total of 23 patients presented with lethargy. At study endpoint, gastrointestinal and neurological symptoms, ketoacidosis and hyperventilation were significantly reduced compared to baseline.

Analysis of data from the safety population showed carglumic acid to have a good safety profile. The number of deaths (*n* = 7) is not unexpected, as OA has a high mortality rate. Mortality rates of approximately 50 % have been reported, however these are related to long-term outcomes and cannot be directly compared with this study [1, 23]. The time between start of an episode and initiation of carglumic acid treatment was similar for the three diagnostic categories. The mean time between start of an episode and initiation of carglumic acid treatment was longer in non-neonates compared to neonates, however the median time was similar. The mean duration of treatment for hyperammonaemia was similar in both neonates and non-neonates. Treatment with carglumic acid was largely initiated at the EMA label recommended dose (100–250 mg/kg). While dose was decreased over time, this decrease was not pronounced. A high first dose of carglumic acid, albeit within the recommended dose range of 100–250 mg/kg, is consistent with the fact that treatment often commences with a loading dose for rapid normalisation of plasma ammonia levels [24] and subsequently a daily dose based on weight and hyperammonaemia severity is administered [13]. There was significant variation between regimens (eg whether the loading dose was high or low) making an analysis by dose quite difficult.

This retrospective study has a number of limitations, including lack of control patients treated only with scavengers, patient data variability, the inability to adjust for confounders (including concomitant drugs and diet) and the small number of patients in analysis subgroups.

We are currently looking into a new follow-up retrospective study designed to compare carglumic acid to ammonia scavengers and/or hemofiltration, provided that an equivalent number of hyperammonemic episodes would be retrieved in the same period of time with inclusion criteria being treatment by ammonia scavengers or hemofiltration. Such a study would still present limitations, as it would be unlikely to match the same numbers of episodes with the same treatment modalities (i.e. 1 or 2 scavengers, with or without hemofiltration).

Also, a prospective study with ammonia scavengers only would raise ethical questions since carglumic acid is now an approved drug for treating hyperammonemia in organic acidemias.

Nevertheless, the current study with its observational design provides valuable information regarding real-life clinical practice and the response of patients who have been treated according to local protocols and subject to the clinical judgement of physicians on a case-by-case basis.

Data from this study are consistent with other published data supporting the use of carglumic acid in PA [8]. Furthermore, Daniotti et al. reported that carglumic acid improves acute therapy and reduces the need for the duration of peritoneal dialysis and haemodialysis [7], and recommended that carglumic acid be initiated as soon as possible, even before confirmation of the exact diagnosis. Indeed, although recent guidelines focus on the use of carglumic acid in NAGS deficiency, the use of carglumic acid in hyperammonaemic patients with suspected NAGS deficiency is also recommended [19].

## Conclusion

The results of this study indicate that carglumic acid at the recommended initial dose of 100–250 mg/kg/day, with or without ammonia scavengers, is an effective and safe treatment to restore normal plasma ammonia concentrations (<60 μmol/L) in episodes of hyperammonaemia occurring during metabolic decompensation in patients with IVA, MMA, and PA.
